# Deletion of the African Swine Fever Virus Gene DP148R Does Not Reduce Virus Replication in Culture but Reduces Virus Virulence in Pigs and Induces High Levels of Protection against Challenge

**DOI:** 10.1128/JVI.01428-17

**Published:** 2017-11-30

**Authors:** Ana L. Reis, Lynnette C. Goatley, Tamara Jabbar, Pedro J. Sanchez-Cordon, Christopher L. Netherton, David A. G. Chapman, Linda K. Dixon

**Affiliations:** The Pirbright Institute, Pirbright, Woking, Surrey, United Kingdom; Northwestern University

**Keywords:** African swine fever, DP148R, virulence, immune response, protection

## Abstract

Many of the approximately 165 proteins encoded by the African swine fever virus (ASFV) genome do not have significant similarity to known proteins and have not been studied experimentally. One such protein is DP148R. We showed that the DP148R gene is transcribed at early times postinfection. Deletion of this gene did not reduce virus replication in macrophages, showing that it is not essential for replication in these cells. However, deletion of this gene from a virulent isolate, Benin 97/1, producing the BeninΔDP148R virus, dramatically reduced the virulence of the virus *in vivo*. All pigs infected with the BeninΔDP148R virus survived infection, showing only transient mild clinical signs soon after immunization. Following challenge with the parental virulent virus, all pigs immunized by the intramuscular route (11/11) and all except one immunized by the intranasal route (5/6) survived. Mild or no clinical signs were observed after challenge. As expected, control nonimmune pigs developed signs of acute African swine fever (ASF). The virus genome and infectious virus were observed soon after immunization, coincident with the onset of clinical signs (∼10^6^ genome copies or 50% tissue culture infective doses/ml). The levels of the virus genome declined over an extended period up to 60 days postimmunization. In contrast, infectious virus was no longer detectable by days 30 to 35. Gamma interferon (IFN-γ) was detected in serum between days 4 and 7 postimmunization, and IFN-γ-producing cells were detected in all pigs analyzed following stimulation of immune lymphocytes with whole virus. ASFV-specific antibodies were first detected from day 10 postimmunization.

**IMPORTANCE** African swine fever (ASF) is endemic in Africa, parts of the Trans Caucasus, the Russian Federation, and several European countries. The lack of a vaccine hinders control. Many of the ASF virus genes lack similarity to known genes and have not been characterized. We have shown that one of these, DP148R, is transcribed early during virus replication in cells and can be deleted from the virus genome without reducing virus replication. The virus with the gene deletion, BeninΔDP148R, caused mild clinical signs in pigs and induced high levels of protection against challenge with the parental virulent virus. Therefore, deletion of this gene can provide a target for the rational development of vaccines.

## INTRODUCTION

African swine fever virus (ASFV) causes a hemorrhagic fever with a high case fatality rate in domestic pigs and wild boars that has a high socioeconomic impact in affected countries. The disease, African swine fever (ASF), is endemic or causes sporadic outbreaks in most sub-Saharan African countries and in Sardinia, Italy. Following the introduction of ASF to Georgia in the Caucasus in 2007, the disease spread to neighboring countries, including the Russian Federation, and from there to Eastern Europe, including the European Union countries Estonia, Latvia, Lithuania, Poland, and the Czech Republic (1–6). The continuing spread of ASF in Europe and Africa threatens further spread, which could have disastrous consequences for the global pork industry. The lack of a vaccine limits control. Improved understanding of virus-host interactions is a high priority to underpin vaccine development.

ASFV is a complex large double-stranded DNA virus with a genome of 173 to 193 kbp, depending on the isolate. The variation in genome length mainly results from the gain or loss of genes belonging to one of five different multigene families (MGFs). The genome contains up to 167 genes, including those encoding enzymes for replication and transcription of the virus genome, which take place predominantly in the cytoplasm. Many genes are not essential for replication in cells, and some of these are known to code for proteins that inhibit host innate and intrinsic defenses (7). Since cells of the monocyte/macrophage lineage are the main targets for virus replication, manipulation of cell function can profoundly influence the host response to infection. ASFV proteins have been demonstrated to inhibit pathways, including type I interferon (IFN) induction and responses, apoptotic cell death pathways, and stress-induced protein synthesis shutoff. The I329L protein acts as a Toll-like receptor 3 (TLR-3) and TLR-4 homologue inhibiting the IFN induction induced through these TLRs (8, 9); members of MGF-360 and MGF-505/530 also inhibit type I IFN induction and increase virus sensitivity to pretreatment with type I IFN (10–12). The A238L protein inhibits the transcriptional activation of immune response genes mediated by several transcription factors that act through the CBP/p300 transcriptional coactivator (13, 14). Several apoptosis inhibitors encoded include a Bcl-2-like protein (A179L), an inhibitor-of-apoptosis (IAP) family protein (A224L), and a C-type lectin (15–19). Conversely, the p54/E183L protein, expressed at late times postinfection, induces apoptosis (20). The DP71L protein recruits protein phosphatase I to dephosphorylate a translation initiation factor, the α subunit of eukaryotic initiation factor 2, and this inhibits global shutoff of protein synthesis and CCAAT/enhancer-binding protein homologous protein (CHOP)-induced apoptosis (21–24).

Deletion of genes from virulent or already attenuated ASFV isolates has identified a number of nonessential genes. The impact of some of these gene deletions on virus replication in cells, virus virulence in pigs, and induction of protection against challenge with virulent virus has been studied (12, 25–28). This has improved understanding of the role of the genes during virus infection and has identified some target genes for construction of live attenuated virus vaccines. Many ASFV genes are of unknown function and do not have significant sequence similarity to other virus or host genes. We evaluated the function of one of these genes of unknown function, DP148R, and showed that deletion of the gene from a virulent isolate did not affect the ability of the virus to replicate in primary macrophages but resulted in reduced virulence in domestic pigs. All pigs, except one immunized intranasally, survived after immunization and subsequent challenge with virulent virus.

## RESULTS

### Timing of DP148R mRNA transcription.

**FIG 1:**
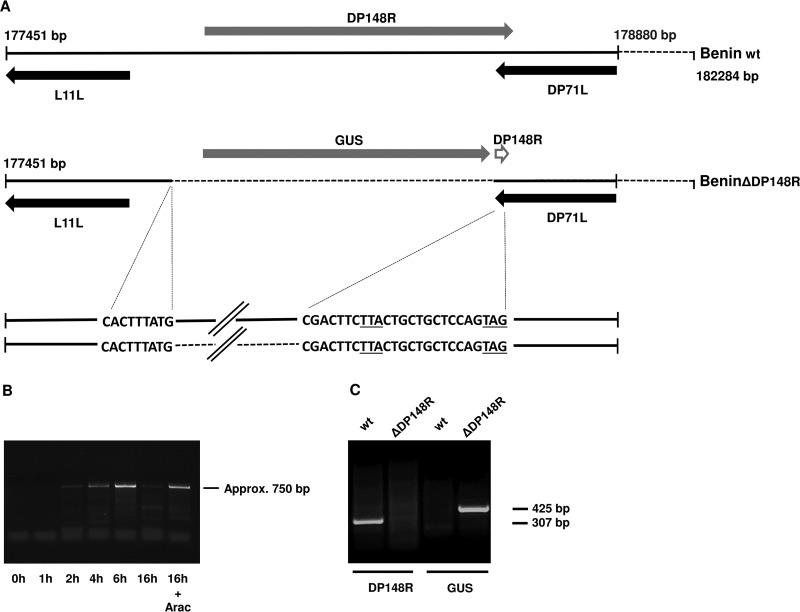
Deletion of DP148R from the Benin 97/1 isolate genome and transcript analysis of the DP148R gene. (A) (Top) Position and direction of reading of the DP148R gene in the wild-type (wt) Benin 97/1 genome relative to those of the upstream and downstream genes. The empty arrow represents the 25 nucleotides from DP148R remaining in the deletion mutant virus. (Bottom) The position of the deletion of the DP148R gene, including the sequence of junction fragments between the inserted reporter GUS gene and the wild-type genome, is indicated. The underlined nucleotides represent the translation stop codons of the DP71L (TTA) and DP148R (TAG) genes. (B) Results of a RACE PCR carried out to measure the level of mRNA transcripts for the DP148R gene at different times following infection of porcine macrophages at a multiplicity of infection with the Benin 97/1 isolate of 1. The time of collection of RNA samples is indicated below each lane. In the lane farthest to the right, the infection was carried out in the presence of AraC, an inhibitor of ASFV DNA replication and late gene expression. The size of the fragment is indicated on the right. (C) PCR analysis of the BeninΔDP148R genome compared to the wild-type Benin 97/1 genome. The left two lanes show a fragment amplified from within the DP148R gene. The two lanes on the right show a fragment amplified from the GUS gene. The sizes of the fragments are indicated on the right.

The DP148R gene is located close to the right end of the ASFV genome and encodes a protein of 254 amino acids in the Benin 97/1 isolate. In previous publications, a similarity to the ASFV MGF-360 genes was reported (29, 30); however, our subsequent analysis has shown that the amino acid sequence has no significant similarity to that of other proteins. The secondary structure is predicted to be predominantly helical, and no evident signal peptide or transmembrane domains could be found (data not shown). The gene is read toward the right end of the virus genome (positions 177915 to 178679) and is flanked by two open reading frames (ORFs) read toward the left genome end. The L11L ORF is at the left of DP148R, and the DP71L ORF is at the right of DP148R (Fig. 1A). An intergenic region of 995 bp is present upstream of the DP148R first ATG codon. The translation stop codon of DP148R overlaps that of DP71L by 5 amino acids. An ASFV predicted transcriptional stop sequence of 7 T residues (31) is present immediately downstream from the translation stop codon of DP148R and on the opposite strand from the DP71L gene between C-terminal residues 6 and 9 of the DP71L gene. Additional potential transcription termination signals for DP148R are present downstream of the DP96R ORF in the intergenic region between DP96R and MGF-360-19R. Predicted transcription termination signals for the DP71L gene are present in the intergenic region between L11L and DP148R.

The timing of transcription of the DP148R gene was analyzed by rapid amplification of cDNA ends (RACE) PCR using RNA collected at different time points after infection of macrophages (Fig. 1B). A band of approximately 750 bp was detected from 2 h postinfection (hpi) and increased in intensity up to a maximum at 6 hpi; however, by 16 hpi the band was no longer visible. The disappearance of the 750-bp band at 16 hpi was blocked in the presence of cytosine β-d-arabinoside (AraC), an inhibitor of virus DNA replication and late ASFV gene transcription. The size of the band was consistent with a transcription start site upstream of the DP148R translation start and in the intergenic region between L11L and DP148R, thus confirming that the transcript detected was the mRNA for DP148R. The temporal expression pattern found, coupled with the effect of AraC, showed that DP148R is an ASFV gene expressed at early times postinfection. The DP71L gene, which is downstream from DP148R, read from the opposite strand and overlapping by 5 amino acids, is expressed at late times postinfection.

### Construction of the Benin 97/1 ΔDP148R deletion mutant (the BeninΔDP148R virus) and testing of replication in macrophages.

Fragments of ∼500 bp from the left and right flanking regions of the DP148R gene from the Benin 97/1 isolate were subcloned into a plasmid upstream and downstream of the β-glucuronidase (GUS) reporter gene under the control of the B646L gene promoter. Following transfection of this plasmid into porcine macrophages infected with the virulent Benin 97/1 isolate, cells infected with recombinant viruses were identified by expression of the GUS gene and purified from wild-type virus. The expected deletion was confirmed by PCR analysis using primers which amplified either the DP148R gene or the GUS gene (Fig. 1C) and by nucleotide sequencing of fragments from the sites of insertion. Figure 1A shows the position of the deletion and the sequence of the sites adjacent to the deletion. The deletion retained the 3′-terminal 25 nucleotides of the DP148R gene, including the translation stop codon, which overlap the 3′ terminus of the DP71L gene, including the translation stop codon.

**FIG 2:**
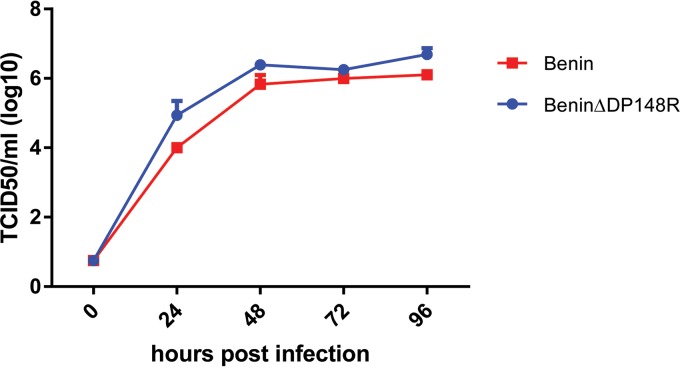
Replication of the BeninΔDP148R virus compared to the wild-type Benin 97/1 strain. Virus titers recovered at different times following infection of porcine macrophages at a multiplicity of infection with the Benin 97/1 or BeninΔDP148R virus of 0.3. The results from a typical experiment are shown. Virus titers in supernatants from duplicate wells were determined by the immunofluorescence assay.

Porcine macrophages were infected with the BeninΔDP148R virus or the parental Benin 97/1 virus at a multiplicity of infection (MOI) of 0.3 to determine if deletion of the DP148R gene affected the ability of the virus to replicate in these cells in culture. At different times postinfection (24, 48, 72, 96 h), cell culture supernatants were harvested and the virus in these supernatants was titrated. The results showed no significant difference between the kinetics and the levels of virus replication of the BeninΔDP148R and parental Benin 97/1 isolates. Virus titers reached a plateau of approximately 10^6^ 50% tissue culture infective doses (TCID_50_)/ml at between 24 and 48 h postinfection. The results show that deletion of the DP148R gene did not significantly alter the ability of the virus to replicate in macrophages in culture (Fig. 2).

### Experimental infections of pigs and challenge with virulent ASFV. (i) Clinical and pathological signs.

**FIG 3:**
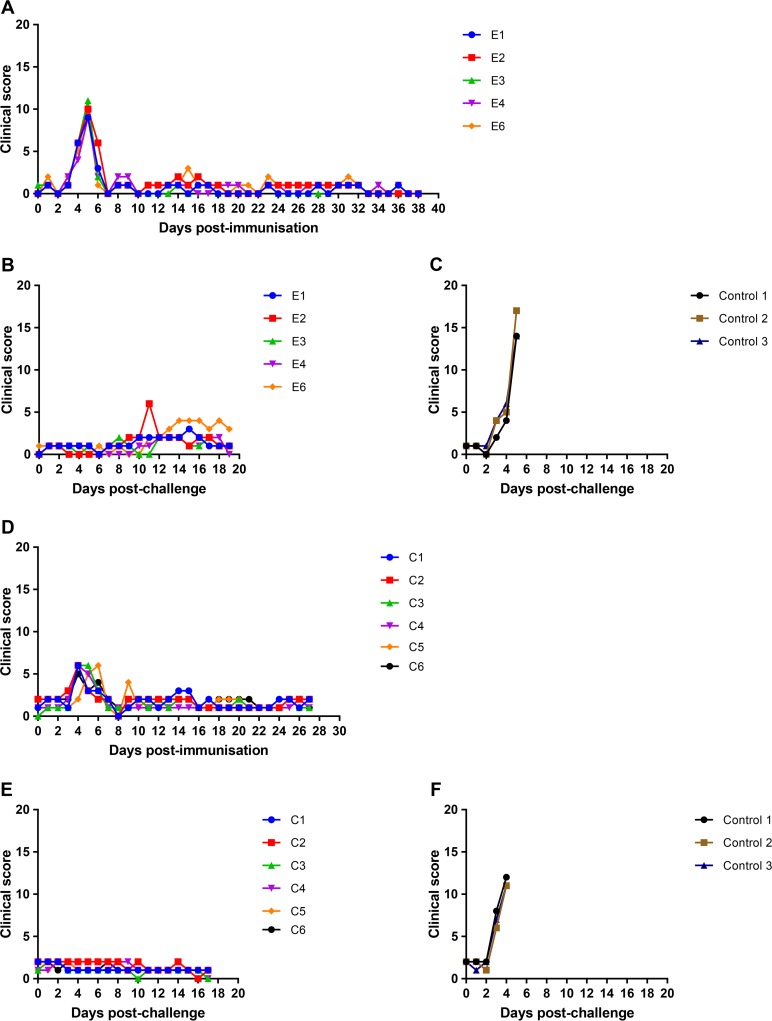
Clinical scores following immunization and boost of pigs with BeninΔDP148R and challenge with Benin 97/1. (A to C) Results from experiment 1; (D to F) results from experiment 2. (A and D) Clinical scores at different days postimmunization i.m.; (B and E) clinical scores at different days after challenge; (C and F) scores for control nonimmune pigs after challenge. Scores for individual pigs are shown in different colors, as indicated in the keys.

**FIG 4:**
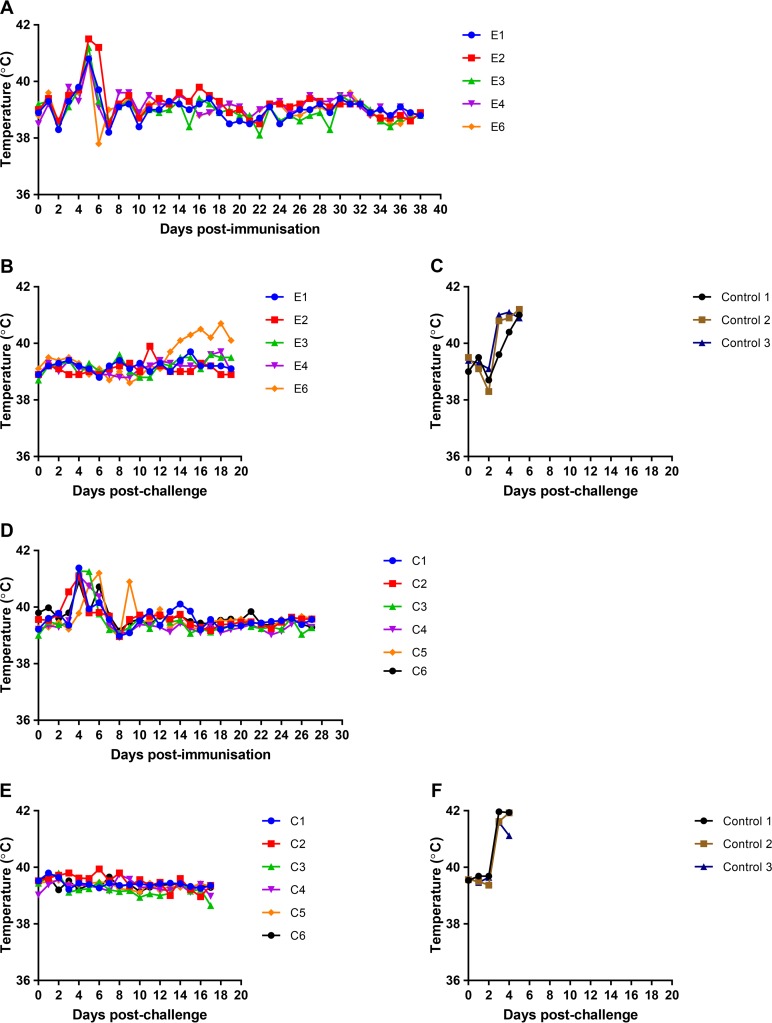
Temperatures following immunization and boost of pigs with BeninΔDP148R and challenge with Benin 97/1. (A to C) Results from experiment 1; (D to F) results from experiment 2. (A and D) Temperatures at different days postimmunization; (B and E) temperatures at different days after challenge; (C and F) values for control nonimmune pigs after challenge. Scores for individual pigs are shown in different colors, as indicated in the keys.

*(a) Experiment 1*. The effect of deleting the DP148R gene on virus pathogenesis in pigs and the effectiveness in inducing protection against lethal challenge were tested. In the first experiment, carried out at The Pirbright Institute, 5 pigs (pigs E1, E2, E3, E4, and E6) were immunized intramuscularly with 10^3^ 50% hemadsorption doses (HAD_50_) of the BeninΔDP148R virus and boosted 21 days later with the same virus. After a further 3 weeks, pigs were challenged intramuscularly (i.m.) with 10^4^ HAD_50_ of virulent parental virus Benin 97/1. In parallel, a group of 3 control pigs was challenged with the same dose of parental virus. Clinical signs were recorded daily from day 0 (immunization). Figure 3A and B show the clinical scores postimmunization and postchallenge, respectively, for the pigs in this group (pigs E1 to E4 and E6). Clinical scores for the nonimmune control group after challenge are shown Fig. 3C. The temperatures of the pigs in group E and the control group are shown in Fig. 4A and B and in Fig. 4C, respectively. All of the pigs immunized with the BeninΔDP148R virus showed clinical signs, including an increased temperature, for 1 or 2 days at between days 4 and 6 postimmunization. No further clinical signs were observed following immunization or boost. After challenge, 3 of the pigs showed no clinical signs, and pig E2 showed an increase in clinical score, including an increased temperature, at day 11. However, pig E6 developed a low fever at day 14 postchallenge that persisted until the end of the experiment. In contrast, the control pigs developed a high fever by day 4 or 5 and other clinical signs consistent with acute ASF. All control pigs were euthanized by day 5 postchallenge, when the humane endpoint was reached. The protected pigs were kept until day 19 postchallenge, which was the end of the experiment.

**FIG 5:**
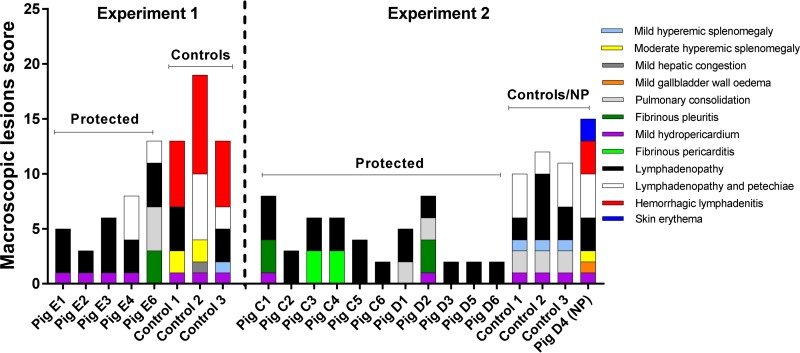
Scoring of macroscopic lesions in pigs at necropsy. The tissues and lesions observed are indicated on the graph by different colors, as indicated in the key. The labels above the graphs shows if the pigs were protected, nonprotected (NP), or control pigs.

Necropsies of control pigs showed gross lesions characteristic of acute ASF (32). Except for mild hydropericardium and generalized lymphadenopathy, the protected pigs did not display significant gross lesions (Fig. 5). It should be noted that a fibrinonecrotic pleuropneumonia affected the caudal lung lobes of pig E6. Histopathological study revealed the existence of hyperemia, pleuritis, and small necrotic foci, along with the presence of neutrophils in alveoli, bronchi, and bronchioles, lesions compatible with respiratory disease caused by bacterial infection. These lesions might explain the transient clinical picture observed in this pig from day 14 postchallenge. Histopathological study also revealed reactive lymphoid follicles and lymph sinuses full of circulating lymphocytes in most of the evaluated lymph nodes from protected pigs. In tonsils, reactive lymphoid follicles were also present. In liver samples, hepatic sinusoids showed circulating cells (monocytes and occasional lymphocytes) along with mildly enlarged Kupffer cells. Cell infiltrates comprising mononuclear cells (lymphocytes and macrophages) in portal spaces, interlobular septa, and sinusoids were also observed.

*(b) Experiment 2*. In the second experiment, carried out at IRTA/CReSA, Barcelona, Spain, 1 group of 6 pigs was immunized intramuscularly with 10^3^ HAD_50_ of the BeninΔDP148R strain (group C) and another group of 6 pigs (group D) was immunized by the intranasal route with 10^3^ HAD_50_. Both groups were boosted 2 weeks later by the same route and with the same dose. Pigs were challenged intramuscularly 2 weeks after the boost with 10^4^ HAD_50_ of virulent parental virus Benin 97/1. A control group of 3 nonimmune pigs was challenged by the same route at the same time as a control group for the inoculum.

As shown in Fig. 3 and 4D and E, the pigs in group C developed a transient fever of 1 or 2 days' duration at between 3 and 6 days postimmunization. No further clinical signs were observed after immunization, boost, or challenge of the pigs in this group; thus, all pigs were protected. All pigs were euthanized by day 17 postchallenge. As expected, pigs in the control group showed clinical signs typical of acute ASF starting from day 3 postchallenge (Fig. 3 and 4F). These signs included increased temperature, lethargy, and a loss of appetite. All were euthanized at the moderate-severity humane endpoint by day 5 postchallenge.

Macroscopic evaluations at necropsy of protected pigs vaccinated i.m. (group C) showed mild gross lesions (Fig. 5). Pig C1 displayed mild hydropericardium along with dorsocaudal chronic pleuritis with pleural adherences, while two pigs (pigs C3 and C4) displayed a chronic pericarditis. In addition, all protected pigs showed generalized lymphadenopathy. Histopathological evaluation confirmed the chronic pleuritis observed in pig C1. In addition, both pig C1 and pig C2 displayed reactive lymphoid follicles in most of the evaluated lymph nodes and tonsils. Mononuclear cell infiltrates in portal spaces, interlobular septa, and sinusoids in liver samples were also observed.

**FIG 6:**
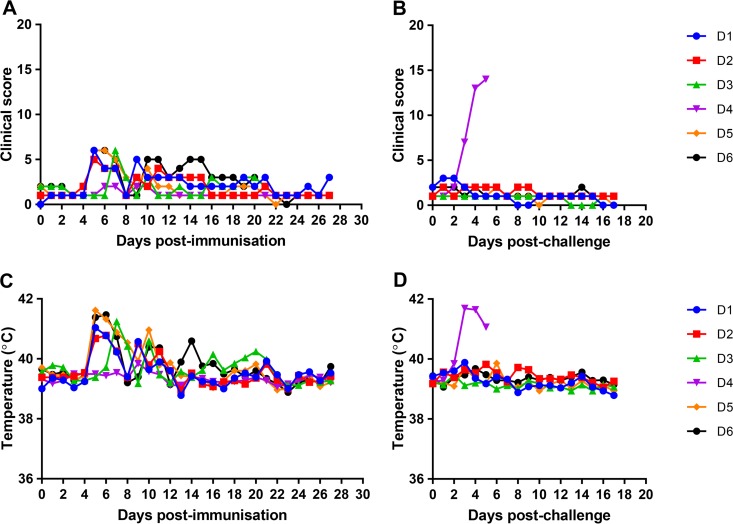
Clinical scores and temperatures in pigs immunized by the intranasal route with BeninΔDP148R and challenged with Benin 97/1. (A) Clinical scores at different days postimmunization and postboost by the intranasal route with BeninΔDP148R; (B) clinical scores postchallenge with Benin 97/1; (C) temperatures at different days postimmunization and postboost; (D) temperatures postchallenge with Benin 97/1. The results for individual pigs are shown in different colors, as indicated in the keys.

In the group of 6 pigs immunized by the intranasal route (group D), five of the pigs had an increased temperature and reduced levels of food consumption postimmunization (Fig. 6A and C). For four of the pigs, these were from day 5 or 6 postimmunization for 2 days, and for one pig (pig D3) these were from day 7. Pig D4 did not show an increase in temperature. After challenge, all pigs except pig D4 were protected and showed no clinical signs. Pig D4 showed clinical signs typical of acute ASFV and was euthanized at day 5 postchallenge when the humane endpoint was reached (Fig. 6B and D). As shown in Fig. 3, the control nonimmune pigs were euthanized by day 5 postchallenge with signs typical of acute ASFV. Thus, all immunized pigs except pig D4 were protected. One possibility was that this pig had not been infected since intranasal (i.n.) delivery was not as effective as i.m. delivery. This was further investigated by measuring ASFV-specific antibody responses using a commercial blocking enzyme-linked immunosorbent assay (ELISA) kit to detect antibodies against p72. This failed to detect an ASFV-specific antibody response (data not shown), confirming that this pig had not been infected either by i.n. inoculation or by contact with the other immunized pigs that were infected.

At necropsy, protected pigs displayed mild gross lesions (Fig. 5). Pig D1 and pig D2 displayed small areas of consolidation in the middle lobes of the lungs. In addition, pig D2 showed dorsocaudal chronic pleuritis with pleural adherences and mild hydropericardium. As in the first experiment, most of the lymph nodes examined in protected pigs showed lymphadenopathy. Histopathological study also revealed the existence of active lymphoid follicles in most of the lymph nodes and tonsils, along with mononuclear cell infiltrates in liver samples.

In both experiments, the clinical scores and temperatures at days 3 and 4 postinoculation were compared between pigs immunized with BeninΔDP148R and control pigs. These were significantly higher (*P* < 0.03) in the animals infected with the parental Benin 97/1 virus (data not shown), confirming that the deletion of DP148R causes viral attenuation *in vivo*.

### (ii) Virus replication in blood.

**FIG 7:**
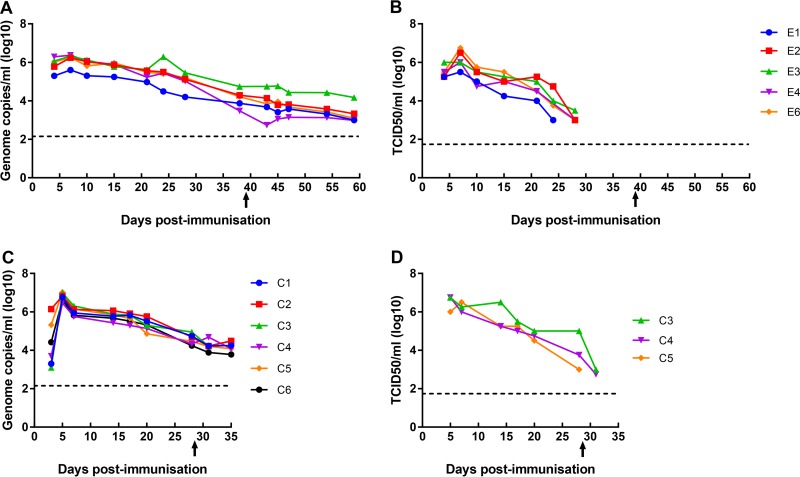
Levels of virus genome and infectious virus detected in blood from pigs immunized with BeninΔDP148R at different days after immunization, boost, and challenge with Benin 97/1. (A and B) Results from experiment 1; (C and D) results from experiment 2. (A and C) Virus genome copy numbers per milliliter (log_10_) estimated by qPCR; (B and D) amount of infectious virus. The results for individual pigs are indicated by different colors, as indicated in the keys. Arrows, time of challenge with Benin 97/1; dashed lines, detection limit of the method used.

*(a) Experiment 1*. The levels of virus in blood samples from experiment 1 were measured by quantitative PCR (qPCR) to detect the virus genome in all immunized and control pigs. Figure 7A shows that in the pigs in group E immunized by the i.m. route, levels of virus genome of between 1 × 10^5^ and 1 × 10^6^ genome copies per ml were detected from day 4 postimmunization. Genome levels reached a peak by day 7 of between 4 × 10^5^ and 2 × 10^6^ genome copies per ml. After this, the number of genome copies per milliliter gradually declined. At termination (day 58 postimmunization), levels of between 1.9 × 10^2^ and 1.5 × 10^4^ genome copies per ml were detected. This was at day 14 postchallenge. However, no increases in virus genome levels were detected following either boost or challenge, indicating that additional virus replication may not have occurred. In control nonimmune pigs, genome levels of up to 10^8^ copies per ml were detected. At day 4 postinoculation (day 4 postchallenge for control pigs or day 4 postimmunization for pigs immunized with BeninΔDP148R), genome copy numbers were significantly higher (*P* = 0.0008) in control pigs (data not shown).

The prolonged and gradual decrease in the level of the virus genome detected in blood from immunized pigs suggested that virus may not have been replicating throughout the period but, instead, may have been attached to red blood cells and gradually lost infectivity. A high percentage (>90%) of ASFV is found attached to red blood cells in blood from pigs infected with hemadsorbing isolates (33, 34); this includes the BeninΔDP148R isolate. This binding is mediated by the CD2v/EP402R virus protein, which is expressed on the surface of infected cells and extracellular enveloped virions (35–37). We measured the levels of infectious virus present in whole-blood samples from immunized pigs of group E. The results (Fig. 7B) showed that infectious virus was first detected at day 4 postimmunization and reached a peak at day 7 postimmunization of between 10^5^ and greater than 10^6^ TCID_50_/ml. After day 7, the levels of infectious virus declined and became undetectable by day 28 at the latest. Thus, the levels of infectious virus were similar to the number of genome copies between days 4 and 10 postimmunization, but from day 15 to day 28, the levels of infectious virus detected decreased dramatically compared to the numbers of genome copies. Thus, infectious virus was detected up to 1 week postboost but was not detected postchallenge.

*(b) Experiment 2*. In experiment 2, pigs immunized with BeninΔDP148R at 10^3^ HAD_50_ i.m. (group C) had detectable levels of the virus genome from day 3 postimmunization, reaching a peak at day 5 of between 10^5^ and 10^7^ genome copies per ml (Fig. 7C). The titers of infectious virus in samples from 3 pigs were determined (Fig. 7D). The levels of infectious virus detected were similar to the genome copy numbers at days 5 and 7 postimmunization but decreased below the genome copy numbers after day 28 postimmunization.

In both experiments 1 and 2, the results are consistent with a limited period of virus replication in blood, followed by a gradual loss of infectivity but persistence of the virus genome.

### (iii) ASFV-specific immune responses induced by BeninΔDP148R.

**FIG 8:**
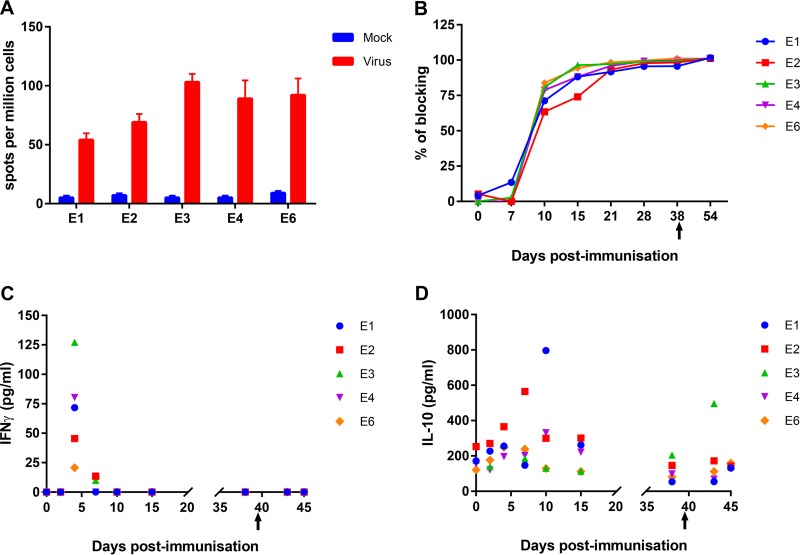
Immune responses detected following immunization with BeninΔDP148R and challenge with Benin 97/1. (A) The numbers of IFN-γ-producing cells detected per million cells following stimulation of PBMC with either medium (mock stimulation) or ASFV strain Benin 97/1. (B) Levels of ASFV-specific antibodies measured using a blocking ELISA on the indicated days postimmunization and postchallenge (*x* axis). The values for individual pigs are shown. (C) Levels of IFN-γ detected in serum by ELISA at different days postimmunization and postchallenge (*x* axis). The results for individual pigs are shown. (D) The levels of IL-10 in serum estimated by ELISA at different days postimmunization and postchallenge (*x* axis). The values for individual pigs are shown. Arrows, time of challenge with Benin 97/1.

Possible mechanisms of protection induced by immunization with BeninΔDP148R were investigated. ASFV-specific cellular and humoral immune responses in animals from experiment 1 were tested by a gamma interferon (IFN-γ)-specific enzyme-linked immunosorbent spot (ELISpot) assay and ELISA, respectively. Peripheral blood mononuclear cells (PBMC) collected prechallenge from pigs in group E (immunized by the i.m. route) were stimulated with virus or a mock inoculum, and the numbers of cells producing IFN-γ were measured by ELISpot assay (Fig. 8A). Mock stimulation induced between 5 and 9 IFN-γ-producing cells per million cells; however, incubation with ASFV induced a 10- to 20-fold increase in the number of IFN-γ-producing cells above the background level. Antibody responses against ASFV major capsid protein p72 in group E were measured using a commercial blocking ELISA. The results (Fig. 8B) showed that low levels of antibodies were detected at day 7 postimmunization in one pig and from day 10 postimmunization in all pigs. The level of antibodies increased by day 15 postimmunization and was maintained in all pigs throughout the experiment. Taken together, the results showed that vaccination with BeninΔDP148R induced both humoral and cellular ASFV-specific responses.

### (iv) Cytokine levels in serum.

The role of cytokines involved in the regulation of the immune response was also explored. The levels of IFN-γ and interleukin-10 (IL-10) in serum were measured at different days postimmunization and postchallenge (group E). A peak in the level of IFN-γ was detected in serum from all pigs at day 4 postimmunization (Fig. 8C), and small amounts (9 to 13 pg per ml) were detected at day 7 but not at other time points. IL-10 was detected in serum from all pigs in group E. The levels remained relatively constant (100 to 300 pg/ml) in pigs E3, E4, and E6 throughout the experiment, including after challenge. In pig E2, an increase to approximately 600 pg/ml was observed at day 7, and in pig E1, an increase to approximately 800 pg/ml was observed at day 10; these increases were followed by a return to lower levels. In pig E3, an increase to about 500 pg/ml was observed at day 43 (day 3 postchallenge) (Fig. 8D).

## DISCUSSION

The lack of information on the function of many ASFV genes hinders the understanding of virus-host interactions and the development of vaccines. Genes that are not essential for virus replication in cells represent potential candidates for deletion so as to construct live attenuated vaccines. In addition, such genes may have roles in evading host defenses. Here we report the phenotype of an ASFV mutant with a deletion of a gene that encodes a protein, DP148R, without significant similarity to other known proteins. Although previously suggested to be a member of MGF-360, we found no significant similarity to genes in this family. We showed that deletion of the DP148R gene from the virulent Benin 97/1 isolate does not reduce replication of the virus in porcine macrophages, suggesting that the encoded protein is not essential. We determined the kinetics of mRNA transcription and showed that the DP148R gene is expressed early in infection.

To determine if the DP148R gene has an important role during virus replication in pigs, we infected animals and observed the development of clinical signs. These experiments showed that deletion of this gene dramatically reduced the pathogenesis of the virus in pigs. Two experiments were carried out. In the first experiment, virus was delivered by the intramuscular route at a dose of 10^3^ HAD_50_, and in the second experiment, one group of pigs was immunized by the intramuscular route and one was immunized by the intranasal route at a dose of 10^3^ HAD_50_. In these experiments, 16 of the immunized pigs had an increased temperature, as well as lethargy and a loss of appetite, for 1 or 2 days. The timing of this increase in clinical signs was more variable for those immunized by the intranasal route than those immunized by the intramuscular route, possibly because the timing of peak virus replication was less synchronized. One pig (pig D4) immunized intranasally did not have an increased temperature but may not have been vaccinated since we did not detect an ASFV-specific antibody response in this pig. We have previously shown that intranasal immunization with a live attenuated vaccine can induce good levels of protection (38). However, this method of immunization may be less reliable, as shown here, and may require higher doses. The obvious advantage of stimulating the local mucosal immunity merits further investigations on this method of vaccine delivery.

No further clinical signs were observed postimmunization or postboost. Thus, all the pigs survived the infection with BeninΔDP148R with a short period of mild clinical signs. All the pigs immunized with BeninΔDP148R survived challenge with virulent parental virus Benin 97/1, with the exception of pig D4, which did not appear to have been infected by BeninΔDP148R. All but 2 of the 16 pigs did not show clinical signs after challenge. Pig E2 showed a transient increase in temperature on day 1, whereas pig E6 had a slightly elevated temperature. At necropsy, the surviving pigs did not show signs associated with acute ASFV infection. The mild chronic cardiorespiratory lesions observed in some protected pigs at termination may have been associated with transient immune suppression predisposing the appearance of opportunistic infections. Histologic evaluation of lymphoid organs taken from protected pigs at termination showed an active immune system without morphological changes that might indicate impaired immune functions.

Measurement of the levels of the virus genome in blood from pigs from two groups showed that all had a reduced level (∼10^6^ genome copies per ml) at day 5 postimmunization compared to the control animals at day 5 postchallenge. The levels of the virus genome declined after this time, but it was detectable over an extended period. Extracellular ASFV particles are known to bind to the surface of red blood cells, and this is mediated by the CD2v protein (35–37). In previous studies, greater than 90% of the virus has been found to be associated with the red blood cell fraction in blood (33, 34). Therefore, we suspected that the extended persistence of the virus genome may result from virus bound to red blood cells, which may lose infectivity over time, rather than the presence of continuously replicating virus. At day 5 postimmunization, the levels of infectious virus were similar to the genome levels, but the amount of infectious virus decreased more rapidly than the amount of the virus genome, and after days 30 to 35, infectious virus was no longer detectable. These results are consistent with our hypothesis, although further experiments are needed to confirm this.

We were interested to determine the possible mechanisms of protection induced by BeninΔDP148R. We tested the responses of lymphocytes collected prechallenge from BeninΔDP148R-immunized pigs in the first experiment to ASFV stimulation using an IFN-γ-specific ELISpot assay. The results showed that IFN-γ-producing cells were induced in all of the pigs tested (pigs E1, E2, E3, E4, and E6). Some correlation between levels of protection and the numbers of IFN-γ-producing cells detected following virus stimulation has been observed previously, but this has not been observed in all studies (12, 39–41). In addition, the protection induced by natural attenuated ASFV isolate OURT88/3 was shown to require CD8^+^ T cells, since depletion of this cell subset abrogated protection (42). The results are consistent with ASFV-specific IFN-γ-producing cells having a role in protection, although other mechanisms cannot be excluded. All pigs from the first experiment had high levels of ASFV-specific antibodies, which were detected from day 10 postimmunization and maintained throughout the experiment. A role for antibodies in protection is suggested by previous experiments showing that the passive transfer of serum from immune to naive pigs could induce partial protection (43). However, the mechanism by which antibodies may protect is not clear, since detection of neutralizing antibodies has been variable (44–46). IFN-γ was detected in serum at day 4 postimmunization but not at other times. A similar increase in serum IFN-γ levels was observed at this time postimmunization of pigs with a different deletion mutant, BeninΔMGF (12). These increases in IFN-γ levels probably resulted from secretion from NK or NKT cells, since they likely occurred before the development of specific T cells. The induction of high levels of NK cells has previously been correlated with protection (47). IFN-γ plays an important role in establishing an adaptive immune response so is likely to have a positive influence on protection. The levels of IL-10 in serum remained relatively unchanged, below 400 pg/ml, throughout immunization and challenge, except in 3 pigs that showed a transient increase at 7 or 10 days postimmunization and 1 pig that showed an increase shortly after challenge (day 43). In our previous experiments, we showed an association between the protection induced by the OURT88/3 strain and lower levels of IL-10 in serum postchallenge. Since IL-10 can act to suppress innate and adaptive responses, a lower level of this cytokine may favor the induction of immune responses correlating with protection (38). However, in other studies in which pigs were infected with a moderately virulent isolate, an increase in IL-10 levels in serum correlated with survival of the pigs (48).

Our results show that the DP148R gene is not essential for ASFV replication in macrophages and that deletion of the gene does not reduce the level of virus replication. However, deletion of the gene dramatically reduced virus virulence, since all pigs infected with BeninΔDP148R survived and were protected against challenge with the virulent parental virus. The early expression of DP148R during infection of cells indicates that the protein encoded by the DP148R gene may play a role in evading the host's defenses, although the function of DP148R is not known. ASFV gene deletions, for example, deletion of genes belonging to MGF-360 and MGF-505/530, have previously been shown to induce higher type I IFN responses in infected macrophages, attenuate ASFV infection in pigs, and induce protection against challenge (10, 12). Only a few single gene deletions have resulted in virus attenuation *in vivo*. For example, deletion of the ERV1-ALR homologue 9GL gene affects virion maturation and viral growth in macrophages and attenuates the virus infection in pigs (49). Further research is required to determine the function of DP148R and if it has a role in inhibiting the innate immune system. Our results confirm that deletion of DP148R provides another target for the production of rationally attenuated ASFV vaccine candidates.

## MATERIALS AND METHODS

### Viruses and cells.

The Benin 97/1 isolate was described previously (29). Viruses were cultured in porcine bone marrow (PBM) or alveolar macrophages (PAMs). Titration of virus was carried out by hemadsorption assay (in which the results are presented as the number of HAD_50_ per milliliter) or by immunofluorescence using antibodies against ASFV early protein p30/CP204L.

### Construction of recombinant ASFV BeninΔDP148R.

Right and left genome fragments of approximately 500 bp flanking the DP148R gene were amplified by PCR and cloned into the pLoxPVP72GUSLoxP vector (50) to construct plasmid pΔDP148RGUS. PAMs were infected with the Benin 97/1 isolate and transfected with the pΔDP148RGUS plasmid using the TransIT-LT1 transfection reagent (Mirus Bio, Madison, WI, USA). Recombinant viruses expressing the GUS gene were identified by incubation of infected cells in the presence of 5-bromo-4-chloro-1H-indol-3-yl-β-d-glucopyranosiduronic acid and purified by limiting dilution.

### Analysis of mRNA transcripts from infected macrophages.

Alveolar macrophages were infected with the Benin 97/1 isolate at a multiplicity of infection (MOI) of 1. RNA samples were collected at different times postinfection (p.i.) using an RNeasy minikit (Qiagen, Hilden, Germany). DP148R transcripts were evaluated by RACE (5′ RACE system for rapid amplification of cDNA ends; Invitrogen) according to the manufacturer's instructions. Briefly, 1 μg of RNA was reverse transcribed using the following gene-specific primer 1 (GSP1): GGAGCAGCAGTAAGAAGTC. After purification of the first-strand product, thymine-deoxyribosylthymine (TdT) tailing of the 3′ end of the cDNA was performed. The transcripts were amplified by PCR using a primer (GCTGCGAGCGTAGTTTGG) designed to be specific for the region upstream of GSP1 and the anchor primer provided in the kit.

### Virus growth analysis.

PBMs were infected at a multiplicity of infection of 0.3 with the Benin 97/1 or BeninΔDP148R virus. Cells and supernatants were collected at different times postinfection and subjected to 3 freeze-thaw cycles. Cellular debris was removed by centrifugation, and virus titers were determined by the immunofluorescence assay.

### Pig immunization and challenge.

**FIG 9:**
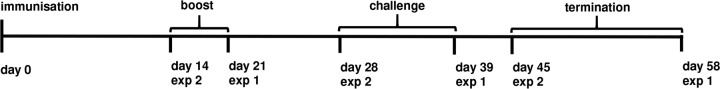
Timeline of animal experiments 1 and 2. The days postimmunization (day 0) when pigs were boosted, challenged, and euthanized are shown.

Experiment 1 was conducted at The Pirbright Institute (Woking, Surrey, United Kingdom), and experiment 2 was conducted at IRTA/CReSA (Barcelona, Spain) according to regulated procedures from the Animals (Scientific Procedures) Act UK 1986. At The Pirbright Institute, female Large White Landrace piglets were obtained from a high-health-status farm. At IRTA/CReSA, crossbred male piglets that were 8 to 9 weeks old and that had been vaccinated against porcine circovirus type 2 (PCV2) were obtained from farms with a high sanitary status where the animals tested negative for porcine respiratory and reproductive syndrome (PRRS) and Aujeszky's disease. In both experiments, pigs were immunized with 10^3^ HAD_50_ BeninΔDP148R and boosted 2 or 3 weeks later with the same dose of virus. Control and immunized pigs were challenged with the virulent parental Benin 97/1 strain 2 to 3 weeks after the boost (Fig. 9).

After immunization (0 dpi), rectal temperatures and clinical signs were monitored daily and scored as described previously (39). Blood (Vacutainer K2 EDTA tubes; Becton Dickinson, UK) and serum (Vacutainer serum tubes; Becton Dickinson, UK) samples were collected from all pigs prior to virus immunization (0 dpi) and at different time points after immunization, boost, and challenge.

Throughout the experiment, vaccinated nonprotected pigs and nonvaccinated control pigs were euthanized at different time points after reaching a specified endpoint, while all protected pigs were euthanized from 17 days postchallenge (dpc). Gross lesions were assessed during necropsies by following scoring methods based on previous standardized protocols (32). During the necropsies, tissue samples were taken and routinely processed for histopathological examination. These included samples of tonsil, lymph nodes, spleen, liver, and lung tissues.

### Quantitative PCR analysis of virus genome copy numbers.

DNA was extracted, using a MagMAX extraction system (Thermo Scientific) and a MagVet universal isolation kit (Life Technologies), from whole peripheral blood that had been collected in EDTA-containing tubes at different days postimmunization. qPCR was carried out on a Stratagene Mx3005P system (Agilent Technologies, Santa Clara, CA, USA) following a protocol modified from that of King et al. (39, 51) using the primers Vp72 sense (CTG CTC ATG GTA TCA ATC TTA TCG A) and Vp72 antisense [GAT ACC ACA AGA TC(AG) GCC GT] and the probe 5′-(6-carboxyfluorescein [FAM])-CCA CGG GAG GAA TAC CAA CCC AGT G-3′-(6-carboxytetramethylrhodamine [TAMRA]).

### Assay of T cell responses.

Peripheral blood mononuclear cells (PBMC) were purified from EDTA-anticoagulated blood using gradient centrifugation. ELISpot assay plates were coated overnight at 4°C with 4 μg/ml P2F6 in 0.5 M carbonate-bicarbonate coating buffer and then washed with phosphate-buffered saline (PBS). Cells were plated in duplicate at two different dilutions, typically, 5 × 10^5^ and 2.5 × 10^5^ per well, in RMPI supplemented with 10% fetal calf serum, 1 mM sodium pyruvate, 50 μM 2-mercaptoethanol, 100 IU/ml penicillin, and 100 μg/ml streptomycin. The cells were then incubated overnight in a final volume of 200 μl with 10^5^ HAD_50_ of Benin 97/1, an equivalent volume of mock inoculum, or 2.5 μg/ml phytohemagglutinin as a positive control. Cells were lysed by incubation for 5 min in water and then washed with PBS. Biotinylated P2C11, followed by streptavidin conjugated to alkaline phosphatase, was used to visualize the spots, which were then counted using an ELISpot assay reader system (Autoimmun Diagnostika GmbH). The number of spots per well was converted into the number of spots per million cells, and the mean for duplicate wells was plotted (52).

### Measurement of antibody responses against ASFV.

The level of antibodies against ASFV-specific protein p72/B646L in serum was measured using a competition ELISA kit (Ingenasa PPA3 Comppac). The percentage of blocking was calculated using the following formula: [(negative-control OD − sample OD)/(negative-control OD − positive-control OD)] × 100, where OD is optical density.

### Measurement of cytokine levels in serum.

The levels of IFN-γ and IL-10 in serum were measured by ELISA according to the manufacturer's recommendations (porcine IL-10 and porcine IFN-γ Quantikine ELISA kit; R&D Systems, Abingdon, UK).

### Statistical analysis.

Statistical analysis was performed using GraphPad Prism (version 6) software. For clinical scores and temperatures, significant differences between groups were determined using two-way analysis of variance followed by Sidak's multiple-comparison test. For differences in genome copy numbers, an unpaired *t* test was used.
